# Sox11 regulates mammary tumour-initiating and metastatic capacity in *Brca1*-deficient mouse mammary tumour cells

**DOI:** 10.1242/dmm.046037

**Published:** 2021-05-10

**Authors:** Siu Man Tsang, Hyojin Kim, Erik Oliemuller, Richard Newman, Naa-Anyima Boateng, Naomi Guppy, Beatrice A. Howard

**Affiliations:** The Breast Cancer Now Toby Robins Research Centre, Division of Breast Cancer Research, The Institute of Cancer Research, London, SW3 6JB, UK

**Keywords:** Sox11, Embryonic mammary progenitor cell, Lineage, Tumour-initiating cell, Mouse mammary tumour

## Abstract

Little is known about the role of Sox11 in the regulation of mammary progenitor cells. Sox11 is expressed by mammary bud epithelial cells during embryonic mammary gland development and is not detected in mammary epithelial cells after birth. As Sox11 is an oncofetal gene, we investigated the effects of reducing Sox11 levels in embryonic mammary progenitor cells and found that Sox11 regulates proliferative state, stem cell activity and lineage marker expression. We also investigated the effect of reducing Sox11 levels in two transplantable *Brca1*-deficient oestrogen receptor-negative mouse mammary tumour cell lines, to assess whether Sox11 regulates similar functions in tumour progenitor cells. When Sox11 levels were reduced in one *Brca1*-deficient mammary tumour cell line that expressed both epithelial and mesenchymal markers, similar effects on proliferation, stem cell activity and expression of lineage markers to those seen in the embryonic mammary progenitor cells were observed. Orthotopic grafting of mammary tumour cells with reduced Sox11 levels led to alterations in tumour-initiating capacity, latency, expression of lineage markers and metastatic burden. Our results support a model in which tumours expressing higher levels of Sox11 have more stem and tumour-initiating cells, and are less proliferative, whereas tumours expressing lower levels of Sox11 become more proliferative and capable of morphogenetic/metastatic growth, similar to what occurs during embryonic mammary developmental progression.

## Abstract (precis)

**Summary:** *Brca1^−/−^* mammary tumours expressing Sox11 at high levels have more stem- and tumour-initiating cells, and are less proliferative, whereas tumours expressing Sox11 at lower levels become more proliferative and are capable of morphogenetic/metastatic growth.

## INTRODUCTION

Embryonic mammary cells are undifferentiated highly plastic progenitor cells that interact with each other to form normal mammary tissues during development (Howard, 2012). The mammary gland continues to develop after birth from descendants of prenatal mammary epithelial cells (Van Keymeulen et al., 2011). Sox11 is an embryonic mammary epithelial marker that is normally silent in postnatal mammary epithelial cells and is expressed in some oestrogen receptor-negative (ER−) and HER2+ breast cancers (Zvelebil et al., 2013). We have recently shown that SOX11 confers features of multipotency, impairs differentiation processes and alters tropism of ER− breast cancer cells to metastatic sites (Oliemuller et al., 2020). Although a number of studies have shown that Sox11 is expressed in mammary stem cells during embryonic mouse mammary development, it is not known whether it has any functional role in regulating normal embryonic mammary progenitor cells (Chung et al., 2019; Makarem et al., 2013; Wuidart et al., 2018; Zvelebil et al., 2013).

Furthermore, it has not been established what role Sox11 plays during mouse mammary tumourigenesis. *Sox11* is expressed at low to moderate levels in basal mammary tumour subtypes in mouse models of breast cancer, including the C3(1)-Tag model and *Brca1^−/−^p53±* model (Zhu et al., 2011). *Sox11* levels increase during tumour progression in some mouse models of breast cancer (Brock et al., 2014), raising the possibility that Sox11 confers embryonic-like traits to mammary tumour cells and promotes tumour progression.

Results from our previous studies have suggested that expression of different levels of SOX11 in normal breast and breast cancer cells leads to distinct phenotypes and behaviour of both normal breast stem cells and breast cancer stem cells (Oliemuller et al., 2017). To further investigate the effect of Sox11 on mouse mammary progenitor cells, we modulated Sox11 levels in embryonic mouse mammary progenitor cells and in transplantable mouse mammary tumour cells, and found that Sox11 influences embryonic mammary progenitor cells and mammary tumour phenotypes in a similar manner.

## RESULTS

### Sox11 is expressed during early stages of embryonic mammary gland development

**Fig. 1.**
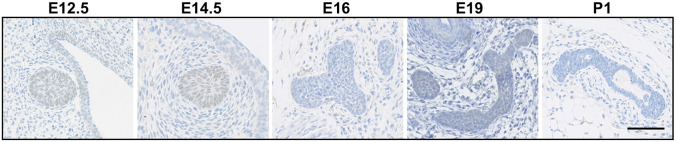
**Sox11 is expressed during early stages of embryonic mammary gland development.** Representative images of Sox11 immunohistochemistry at different development stages (E12.5 to P1) of BALB/c embryos. Scale bar: 100 μm.

Embryonic mammary epithelial cells are initially multipotent before undergoing lineage restriction, which occurs before birth (Lilja et al., 2018; Wuidart et al., 2018). Sox11 expression has been reported previously during embryonic mouse mammary development at embryonic day (E)12.5 stage in the C57BL/6 strain, when most embryonic mammary epithelial cells are thought to be multipotent (Zvelebil et al., 2013). Using immunohistochemistry, we sought to determine when Sox11 protein expression can be detected between E12.5 stage and birth. We found that Sox11 was expressed in mammary epithelial progenitor cells between E12.5 and E14.5 stages in the Balb/c strain (Fig. 1). By E16.0 stage, Sox11 was no longer detected. No Sox11 was detected at later embryonic stages or in postnatal day (P)1 mammary tissues.

### Sox11 regulates stem cell proliferation, survival and lineage marker status of mouse embryonic mammary progenitor cells

**Fig. 2.**
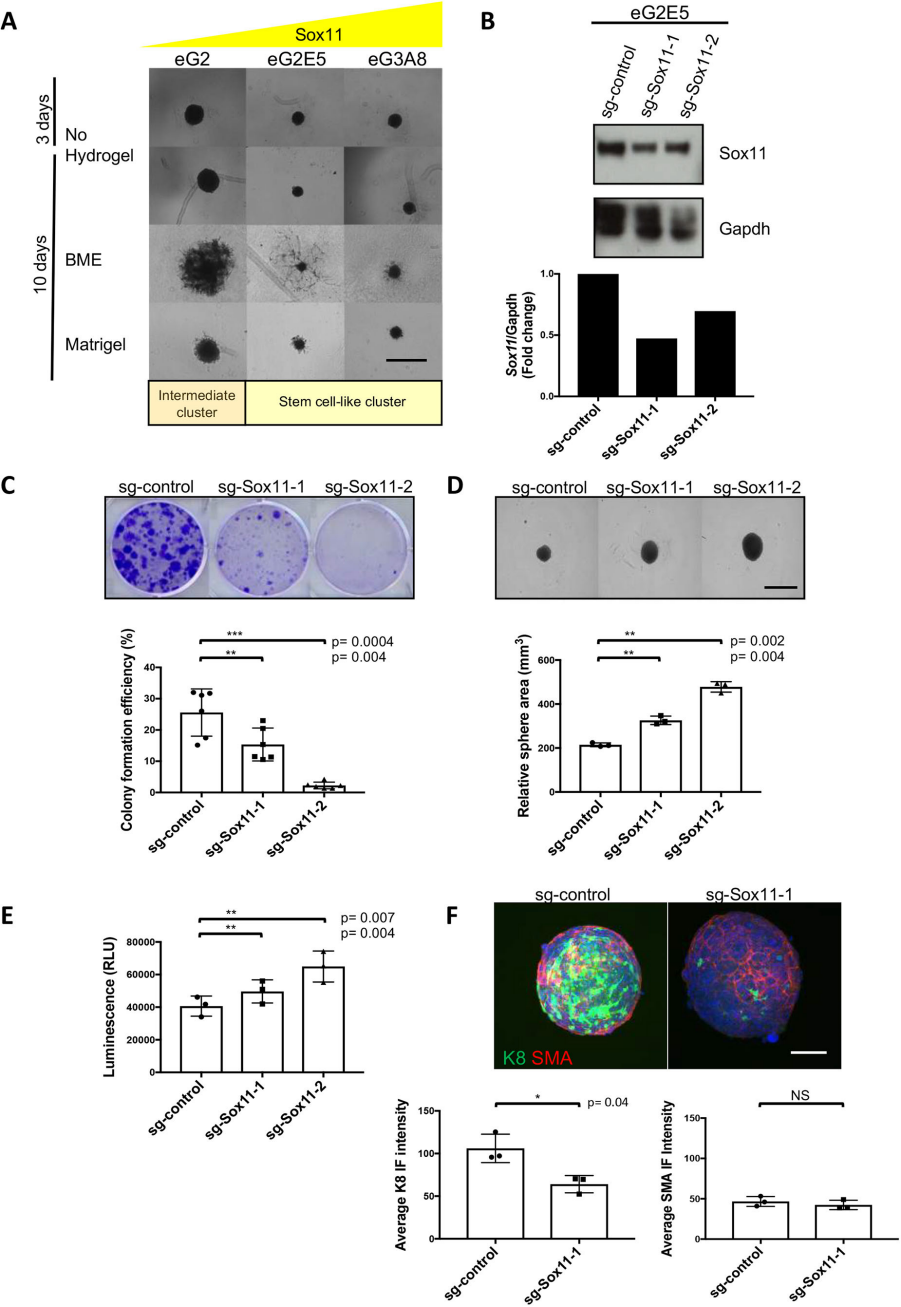
**Sox11 regulates stem cell proliferation, survival and lineage marker status of mouse embryonic mammary progenitor cells.** (A) Embryonic mammary progenitor cells with varying levels of Sox11 were grown in low attachment 96-well plates for 3 days before the addition of Matrigel or BME. Image analysis of mammospheres were performed at 10 days. *N*=3 independent experiments. Three to four replicates per group per experiment. (B) Expression of Sox11 in eG2E5 ‘stem-like cells’ after CRISPR/Cas9 gene editing. Western blot showing knockdown of Sox11 with guide #1 and guide #2. (C) Colony formation assay results from eG2E5 cells when Sox11 levels are reduced. *N*=3 independent experiments. Two replicates per group per experiment. (D) Sphere formation assay after knockdown of Sox11. eG2E5 cells were grown in low attachment 96-well plates for 10 days before image analysis. *N*=3 independent experiments. Eight replicates per group per experiment. (E) CellTiter-Glo results from eG2E5 cells when Sox11 levels are reduced. *N*=3 independent experiments. Eight replicates per group per experiment. (F) Immunofluorescence (IF) analysis of K8 in sg-Sox11-1 compared to sg-control spheres of eG2E5 cells. Sections were projected on one plane with maximum intensity (*z* stack). Green, K8; red, SMA. Magnification 20×. *N*=3 independent experiments. Average of three replicates per group per experiment. Data are mean±s.d. Paired two-tailed Student's *t*-test. Scale bars: 1000 μm (A,D); 200 μm (F). NS, not significant; RLU, relative light unit.

We used embryonic mammary progenitor cell (eMPC) lines to assess the functional role of Sox11 by using CRISPR/Cas9 targeting (Fig. S1A). eMPC lines were described in our previous study (Kogata et al., 2018). eMPC lines eG2E5 and eG3A8 are more stem cell-like based on gene expression and cluster analysis, and express higher levels of *Sox11* than eG2 (Fig. S1B), an intermediate cluster member, which clusters close to both the stem cell-like and epithelial clusters (Kogata et al., 2018). Karyotype analysis of 20 G-banded metaphase spreads showed that eMPC lines are polyploid (data not shown). Cells with higher levels of Sox11 form small compact spheres, whereas cells with lower levels of Sox11 form larger spheres (Fig. 2A). When grown with basement membrane extract (BME) or Matrigel, sphere size increased in eG2, but not in the stem cell-like cell lines eG2E5 and eG3A8, suggesting that eMPC cells with higher levels of Sox11 could have a lower morphogenetic or proliferative capacity in these growth conditions. CRISPR/Cas9 targeting was used to knock down *Sox11* in three eMPC lines: eG2 (intermediate cluster) and eG2E5 and eG3A8 (stem cell-like cluster). Variable levels of reduction of Sox11 expression were achieved. Incomplete targeting of *Sox11* is likely due to polyploidy of the cell lines (Fig. 2B; Fig. S1C). We assessed the effects of reducing Sox11 at the cell population level for all subsequent experiments, as single cell-derived clones can exhibit significant clonal variability and may not be truly representative of the general population.

eG2E5, a member of the stem cell-like cluster, was selected for more extensive characterisation of the function of Sox11 in mammary progenitor/stem cells as the other stem-cell like clone eG3A8 grew very slowly *in vitro*. Western blots showed targeting efficiency of *Sox11* in eG2E5 at the population level with reduction of *Sox11* levels of 60% with guide #1 and 30% with guide #2 (Fig. 2B). This result was further confirmed by qPCR, which showed a similar pattern compared to the western blot data, with guide #1 achieving more effective knockdown than guide #2 (Fig. S2A). Knockdown of *Sox11* in eG2E5 cells led to reduced two-dimensional colony-forming efficiency (Fig. 2C), and the relative sphere area increased and a greater number of viable cells were detected compared to controls (Fig. 2D,E). This is consistent with the phenotypes observed in parental eMPCs, in which cells with lower levels of *Sox11* form larger spheres (Fig. 2A). Knockdown of *Sox11* in eG2E5 cells did not result in significant morphological growth in the presence of Matrigel (Fig. S2B). Reducing Sox11 levels in eG2 cells led to modest increases in sphere area and enhanced morphological growth in Matrigel (Fig. S2C-F), but had little effect on the growth of eG3A8 spheres. Furthermore, spheres formed from eG2E5 cells with reduced levels of Sox11 displayed a significant reduction of cells expressing keratin 8 (K8, also known as Krt8), a luminal marker, whereas the frequency of cells expressing SMA (also known as Acta2), a basal marker, did not change when assessed by immunofluorescence (Fig. 2F). Immunofluorescence did not detect keratin 14 (K14, also known as Krt14) expression in eMPC spheres (data not shown).

### Sox11 regulates stem cell activity and lineage status of mouse mammary tumour cells

We used transplantable mouse mammary tumour lines to study the effect of reducing *Sox11* levels on stem cell activity. Two mammary tumour cell lines derived from *Brca1*f/f/*p53*±/BLG-Cre mammary tumours (Molyneux et al., 2010), *Brca1.*3 and *Brca1.*1516, express low levels of *Sox11* in comparison to C2C12, an immortalised mouse myoblast cell line (Fig. S1B). *Brca1.*3 and *Brca1.*1516 cells have *Brca1* deleted from the mammary epithelial luminal progenitor cells and produce tumours that are phenotypically similar to human *BRCA1^−/−^* breast cancers (Molyneux et al., 2010). *Brca1.*3 cells display a luminal progenitor-like morphology when cultured in Matrigel (Fig. S3A) and have a longer latency of ∼8 weeks to tumour formation when xenografted orthotopically (data not shown). In contrast, *Brca1.*1516 cells form spheroids with numerous projections when cultured in Matrigel (Fig. S3A) and have a latency of ∼4 weeks (data not shown).

**Fig. 3.**
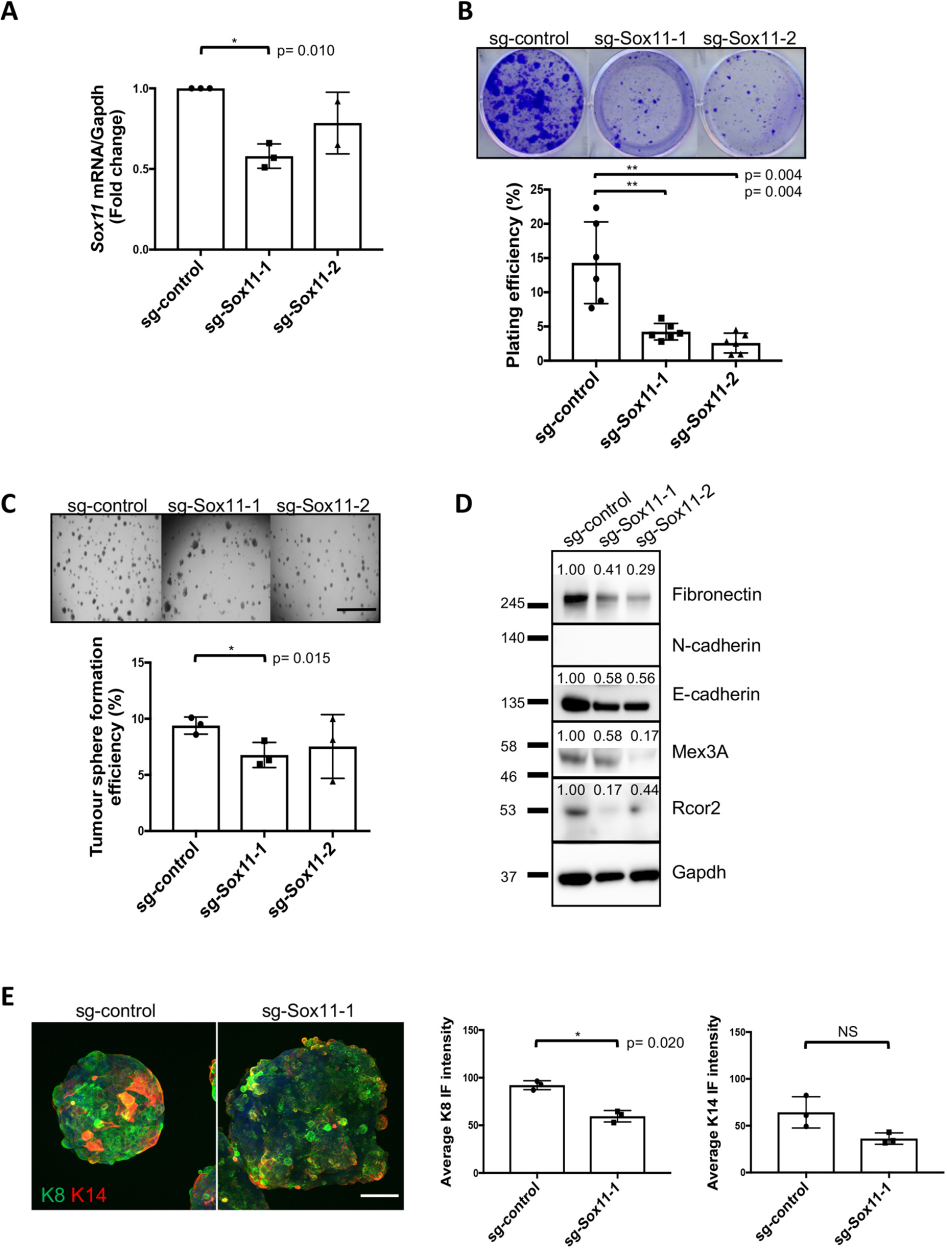
**Sox11 regulates stem cell activity and lineage status of *Brca1*.3 mouse mammary tumour cells.** (A) Expression of Sox11 in *Brca1*.3 cells after CRISPR/Cas9 gene editing. qRT-PCR showing knockdown of Sox11 with guide #1 and guide #2 compared to sg-control. *N*=3 independent experiments. Three replicates per group per experiment. (B) Colony formation assay results from *Brca1*.3 cells when Sox11 levels are reduced. *N*=3 independent experiments. Two replicates per group per experiment. (C) Sphere-forming capacity of sg-control and sg-Sox11-1 cells. Single cells were grown in the presence of 4% Matrigel for 10 days before image analysis. *N*=3 independent experiments. Six replicates per group per experiment. (D) Western blot showing fibronectin, N-cadherin, E-cadherin, Mex3A and Rcor2 after knockdown of Sox11 compared to sg-control. *N*=3 independent experiments. (E) Immunofluorescence (IF) analysis of K8 and K14 in *Brca1*.3 cells. Sections were projected on one plane with maximum intensity (*z* stack). K8 in sg-Sox11-1 spheres compared to sg-control spheres. Green, K8; red, K14. Magnification 20×. *N*=3 independent experiments. Three to five replicates per group per experiment. Data are mean±s.d. Paired two-tailed Student's *t*-test. Scale bars: 2000 μm (C); 200 μm (E). NS, not significant.

Both *Brca1^−\−^* cell lines were targeted with guides for *Sox11* and non-targeting controls. qPCR showed targeting efficiency of *Sox11* in *Brca1*.3 at the population level with reduction of *Sox11* levels of 40% with guide #1 and 20% with guide #2 (Fig. 3A). Analysis of the targeting efficacy showed no evidence of CRISPR/Cas9-mediated changes in three of the most likely predicted off-target sites (data not shown). We found that reducing *Sox11* levels in *Brca1.*3 cells led to reduced colony formation in two dimensions and reduced tumour sphere-forming efficiency in the presence of Matrigel when compared to control spheroids (Fig. 3B,C). When *Sox11* levels were reduced in the cell population (guide #1 and #2), loss of expression of fibronectin (also known as fibronectin 1) and E-cadherin (also known as cadherin 1) were observed (Fig. 3D). N-cadherin (also known as cadherin 2) expression was not detected in *Brca1.*3 cells. Expression of two potential downstream effectors of SOX11 signalling we recently identified in breast cancers cells, Mex3a and Rcor2, which have links to stem cell proliferation and reprogramming, respectively, were also substantially reduced in *Brca1.*3 cells with reduced *Sox11* levels (Fig. 3D) (Barriga et al., 2017; Pereira et al., 2013; Yang et al., 2011). Immunofluorescence analysis detected a substantial reduction in the expression of the luminal marker keratin 8, but not the basal marker keratin 14, in spheroids when *Sox11* levels were reduced (guide #1) (Fig. 3E).

**Fig. 4.**
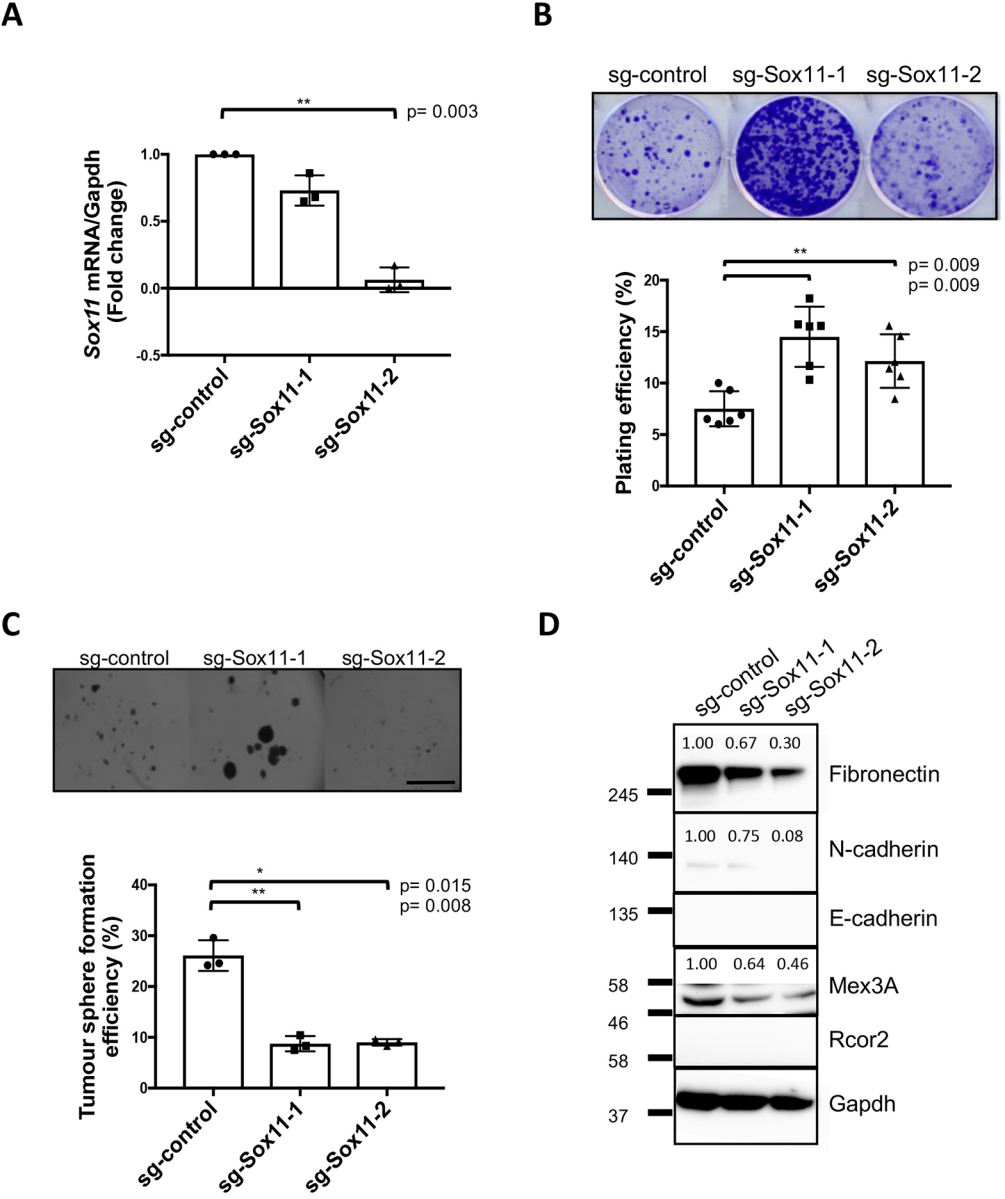
**Sox11 regulates stem cell activity and lineage status of *Brca1*.1516 mouse mammary tumour cells**. (A) Expression of Sox11 in *Brca1*.1516 cells after CRISPR/Cas9 gene editing. qRT-PCR showing knockdown of Sox11 with guide #1 and guide #2 compared to sg-control. *N*=3 independent experiments. Three replicates per group per experiment. (B) Colony formation assay results from *Brca1*.1516 cells when Sox11 levels were reduced. *N*=3 independent experiments. Two replicates per group per experiment. (C) Sphere-forming capacity of sg-control and sg-Sox11-1 cells. Single cells were grown in the presence of 4% Matrigel for 10 days before image analysis. *N*=3 independent experiments. Two replicates per group per experiment. Scale bar: 2000 μm. (D) Western blot showing fibronectin, N-cadherin, E-cadherin, Mex3A and Rcor2 after knockdown of Sox11 (guide #1) compared to sg-control. *N*=3 independent experiments. Data are mean±s.d. Paired two-tailed Student's *t*-test.

We next knocked down *Sox11* in *Brca1*.1516 at the population level and observed a 30% reduction of *Sox11* levels with guide #1 and 95% with guide #2 (Fig. 4A). We found that reducing *Sox11* levels in *Brca1.*1516 cells led to alterations in cell phenotypes, even with just a 30% reduction in *Sox11* levels, and the near ablation of *Sox11* expression led to even more pronounced cell spreading and stellate morphology (Fig. S3B). Colony formation ability increased, whereas sphere-forming capacity decreased, when *Sox11* levels were reduced (Fig. 4B,C). Reductions in fibronectin, N-cadherin, and Mex3A levels were observed when *Sox11* levels were reduced and correlated with the amount of *Sox11* knockdown (Fig. 4D). We were unable to detect the expression of E-cadherin in *Brca1.*1516 cells by western blotting or to detect K8 expression in spheroids by immunofluorescence (data not shown), which suggests that *Brca1.*1516 cells are in a more mesenchymal state compared to *Brca1.*3 cells.

### Sox11 regulates the proliferation of mouse mammary tumours in *Brca1.*3 cells

**Fig. 5.**
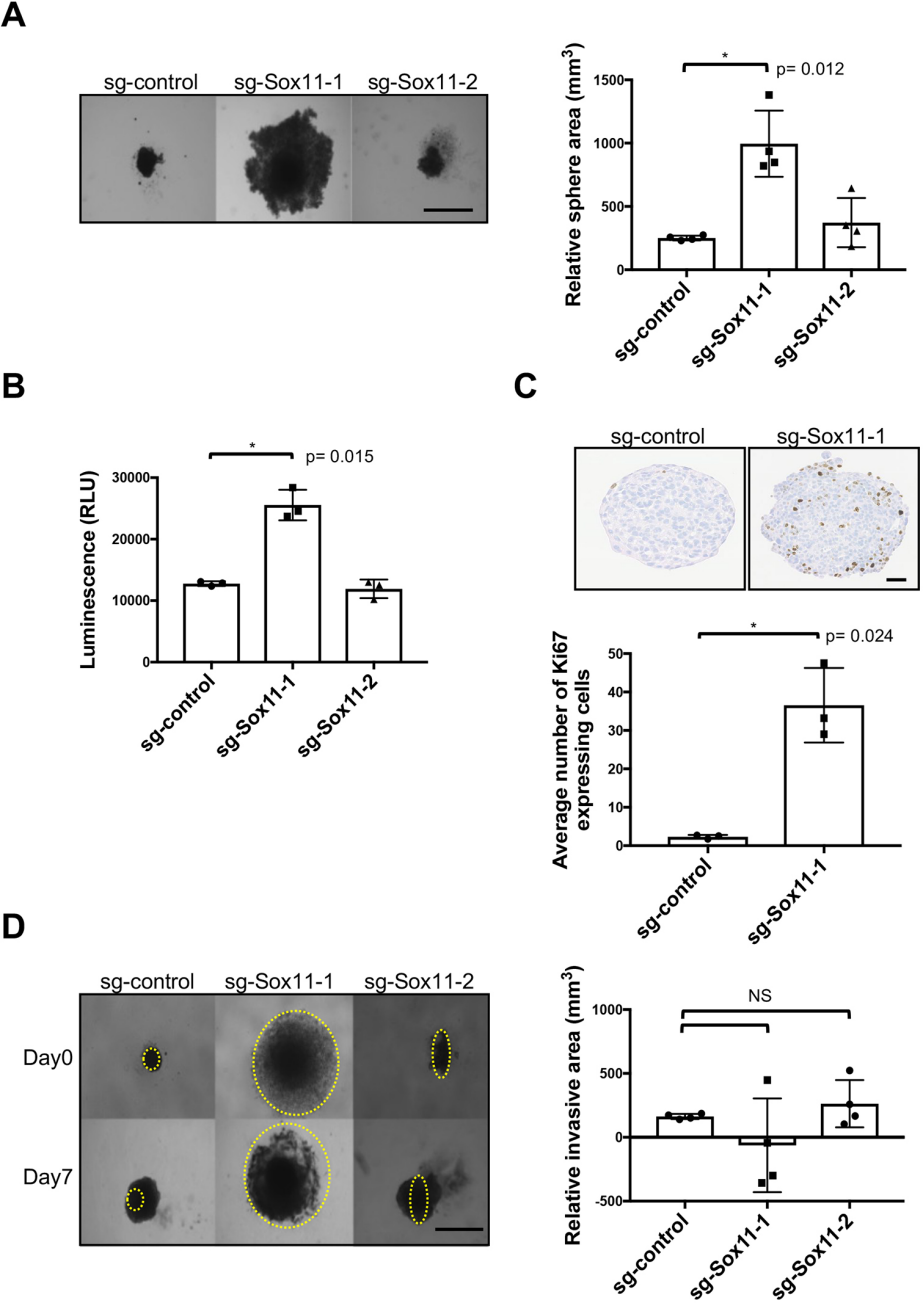
**Sox11 regulates proliferative status of *Brca1*.3 tumour cells.** (A) Sphere formation assay after knockdown of Sox11. *Brca1.*3 cells were grown in low attachment 96-well plates for 10 days before image analysis of relative sphere area. *N*=4 independent experiments. Three replicates per group per experiment. (B) Cell viability as determined by CellTiter-Glo assay. *N*=3 independent experiments. Seven replicates per group per experiment. (C) Quantification of Ki67-expressing cells compared to control in spheroids. *N*=3 independent experiments. Two to four replicates per group per experiment. (D) Three-dimensional invasion assay after knockdown of Sox11. *Brca1.*3 cells were grown in low attachment 96-well plates for 7 days before the addition of Matrigel for a further 7 days before image analysis. Relative invasive area was calculated by subtracting the sphere area of day 7 from day 14. *N*=4 independent experiments. Three replicates per group per experiment. Data are mean±s.d. Paired two-tailed Student's *t*-test. Scale bars: 1000 μm (A,D); 200 μm (C). NS, not significant. RLU, relative light unit.

*Sox11*-deficient *Brca1.*3 spheroids grown on low attachment plates greatly increased in size, resulting in a less compact and disorganised spheroids compared to controls, suggesting that these cells may have been released from a more quiescent state (Fig. 5A). A greater number of viable cells were detected when *Sox11* levels were reduced in *Brca1.*3 cells by guide #1 (Fig. 5B). Ki67^+^ cells were substantially more abundant in spheroids with reduced *Sox11* levels (Fig. 5C), consistent with a role for Sox11 in keeping tumour cells in a non-proliferative state when expressed at higher levels. However, *Sox11*-deficient *Brca1.*3 spheroids did not show any obvious signs of invasion in Matrigel invasion assays, but rather gave rise to more compact spheres compared to controls (Fig. 5D). A small reduction in the cell viability of *Sox11*-deficient *Brca1.*1516 cells was detected (Fig. S3C), but no changes in relative sphere area or frequency of Ki67^+^ cells were detected when *Sox11* levels were ablated in the *Brca1.*1516 spheroids (Fig. S3D,E). Similarly, *Sox11*-deficient *Brca1.*1516 spheroids did not show any signs of altered invasive capacity when assessed by Matrigel invasion assays (Fig. S3F).

### Sox11 regulates tumour growth, lineage status and metastasis *in vivo*

**Fig. 6.**
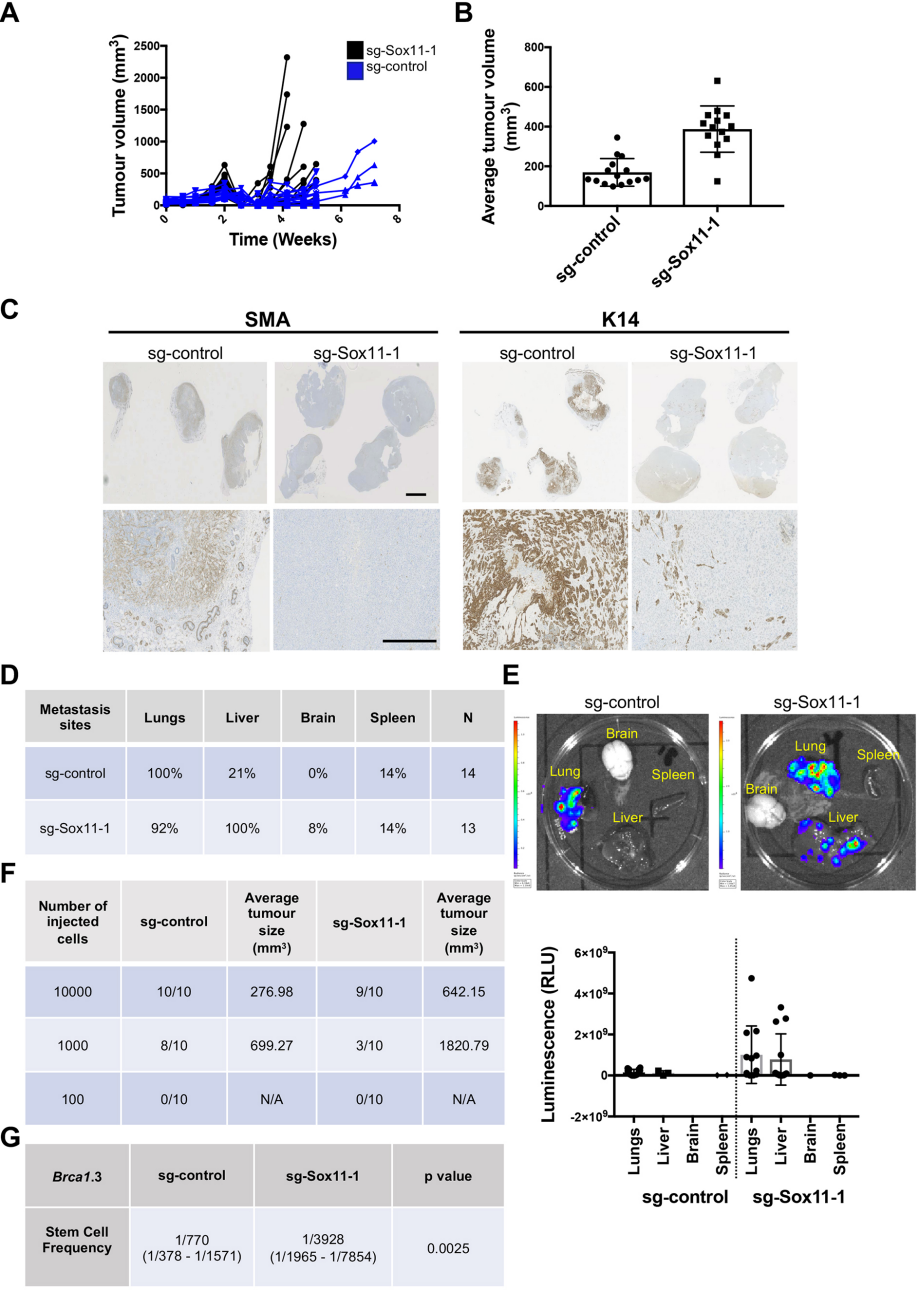
**Sox11 regulates tumour growth, lineage status and metastasis of Brca1.3 cells *in vivo*.** (A) Tumour volume curve of the experiment. Fifteen mice per cohort. (B) Bar graph of tumour volumes at 2 weeks post-injection. (C) Immunohistochemistry of primary tumours showing the expression markers SMA and K14 (three samples for sg-control immunohistochemistry for SMA). The others are eight samples per group. Scale bars: 5 mm (top panels) and 1 mm (lower panels). (D) The percentage of metastasis to each distant organ in each cohort. (E) Bioluminescence imaging and measurement (RLU) of secondary organs *ex-vivo* revealed the presence of tumour cells in brain, lung, spleen and liver at study endpoint. *N*=13 and 14 mice per group. (F) Serial dilutions of primary tumour sg-Sox11-1 and sg-control cells were resuspended in Matrigel, transplanted into the left and right inguinal mammary fat pads of NSG mice. *n*=5 mice, ten injected sites per group. (G) *In vivo* limiting dilution assay of Brca.3 sg-Sox11-1 and sg-control cells. Stem cell frequency was calculated using the ELDA software web interface. Data are mean±s.d. RLU, relative light unit.

We orthotopically injected *Brca1.*3 cells with *Sox11* levels reduced by 40% (with guide #1), and cells targeted with the control guide. Larger average tumour volumes (388 mm^3^) were observed in tumours with reduced *Sox11* levels compared to control tumours (170 mm^3^) at 2 weeks post-injection (Fig. 6A,B). Primary sg-control tumours stained by immunohistochemistry displayed a large proportion of cells expressing basal markers K14 and SMA, markers typically expressed in many *Brca^−/−^1* mammary tumours (Molyneux et al., 2010). A reduced number of tumour cells that express Keratin 14 and SMA were detected in tumours with reduced *Sox11* levels compared to controls (Fig. 6C; Fig. S4B). Tumours with reduced *Sox11* levels stained positive for both K14 and SMA in the normal mammary tissues adjacent to the tumour, which provided internal positive controls (Fig. S4A). IVIS imaging was carried out at the study endpoint (when tumours approached or exceeded 750 mm^3^) to assess whether metastatic cells were present at distant sites. Frequencies of metastasis to lungs, spleens and brain were similar in both cohorts. However, liver metastases were detected more frequently in mice that had primary tumours with reduced *Sox11* levels (Fig. 6D). Furthermore, *ex vivo* bioluminescence imaging and H&E staining showed that higher levels of tumour burden were detected in both the lungs and liver in the *Sox11*-deficient cohort (Fig. 6E; Fig. S4C). Larger tumours were also formed in the *Sox11*-deficient cohort in limiting dilution studies (Fig. 6F), and a statistically significant fivefold reduction in stem cell frequency was detected (Fig. 6G). Together, our results suggest that Sox11 enhances mammary tumour-initiating activity and influences metastatic capacity at specific sites.

## DISCUSSION

We show in this study that Sox11 is expressed in mammary bud epithelial cells from E12.5 to E14.5 during embryonic mouse mammary development, when the cells are largely quiescent (Balinsky, 1950; Lee et al., 2011). Our results indicate that Sox11 expression is downregulated at the time during development when unipotency emerges (Lilja et al., 2018; Wuidart et al., 2018), and the cells become proliferative and invade into the underlying tissues (Balinsky, 1950). Embryonic progenitor cells (eMPC) were used to complement studies of Sox11 in mouse models of breast cancer progression and to examine whether similar functions are regulated in normal mammary progenitor cells compared to tumour progenitor cells. We show that reducing *Sox11* levels in the *Brca1*-deficient mammary tumour (*Brca1*.3) has similar effects on proliferation, stem cell activity and expression of lineage markers to those observed in studies using embryonic mammary progenitor cells (eG2M5).

Small-scale single-cell RNA-seq analysis of E14.5 stage mammary epithelial cells showed that most non-proliferating Lgr5^+^ cells express high levels of *Sox11* (Wuidart et al., 2018). eMPC cells eG3A8 and eG2E5, both of which belong to the stem cell-like cluster, express higher levels of *Sox11.* Our results show that spheres with higher levels of *Sox11* are smaller and compact, whereas spheres with lower levels of Sox11 are larger, and have more viable cells and are less spherical. These changes mimic what is observed morphologically as Sox11 levels change during embryonic mammary gland development between E12.5 and E16.0.

We found that reducing Sox11 levels led to decreased colony formation and an increase in both sphere area and cell viability, suggesting that Sox11 regulates mammary stem cell activity and viability of embryonic mammary progenitor cells. eG2 and eG3A8 progenitor cells did not grow sufficiently using standard eMPC *in vitro* culture conditions, so experiments were limited to sphere morphology. Further optimisation of eMPC culture conditions would be required to permit direct comparisons of different clones under identical conditions.

Single-cell RNA-seq has shown that in most developing organs embryonic cells exist in a hybrid epithelial/mesenchymal state; bioinformatic analyses have suggested that *Sox11* positively regulates the expression of mesenchymal markers, including N-cadherin (*Cdh2*) and fibronectin 1 (*Fn1*) in several developing organs, including intestine, liver, lung and skin (Dong et al., 2018). Surprisingly, a dramatic reduction in expression of both fibronectin and epidermal and mammary lineage markers were observed after Sox11 levels were reduced in mammary tumour cells. Additionally, a loss of luminal marker expression was observed in embryonic mammary progenitor cells. Reduction of E-cadherin and basal marker expression was observed in *Brca1^−/−^* tumours when Sox11 levels were reduced. Transcriptional profiling of *Sox11^−/−^* epidermis from developing mouse embryos (E16 stage) detected downregulated expression of genes encoding markers of simple epithelium, including the cytokeratins *K4*, *K8* and *K19* (Miao Q, 2018). This raises the possibility that the reduction in K8, K18 and K14 expression detected in mammary tumours with lower Sox11 levels is due to a lack of activation of an epidermal differentiation programme, and these tumours lack the capacity to form differentiated cells.

Previously, Oliemuller et al. (2017) showed that constitutive expression of low levels of SOX11 using the CMV promoter led to enhanced invasive growth of ductal carcinoma *in situ* lesions. A significantly higher level of SOX11 expression was achieved using a doxycycline (DOX)-inducible EF1A promoter (Oliemuller et al., 2020). DOX-induced expression of SOX11 in DCIS.com cells led to increased expression of markers associated with mesenchymal state, increased size of the ALDH^+^/CD24^+^ CSC population, and increased frequency of cells expressing both luminal (K8) and basal (K14, SMA) lineage markers. Furthermore, small mammary tumours that formed from cells expressing high levels of SOX11 grew out quickly when DOX chow was replaced with normal chow, suggesting that high levels of SOX11 may keep tumours in a non-proliferative state and that proliferation occurs when SOX11 levels were lowered upon DOX withdrawal (Oliemuller et al., 2020).

Our knockdown system in mouse mammary tumour cells shows similar trends in many of the phenotypes we observed when comparing cells expressing higher versus lower levels of SOX11 in human breast cancer models. Interestingly, we observed that varying the levels of *Sox11* in both the mouse and human mammary tumour cells affects metastatic tropism. Higher levels of SOX11 in DCIS.com cells led to increased metastatic burden to the brain in xenograft studies (Oliemuller et al., 2020), whereas in our current study, lower levels of *Sox11* led to shortened tumour latency and increased metastatic burden, particularly to the liver. To understand the underlying mechanisms used by these cells to metastasize to distinct sites will require further studies.

In this study, *Brca1.*3 cells from the control cohort displayed enhanced mammary tumour-initiating activity and cells in tumour spheroids appeared to be in a non-proliferative state compared to cells with lower *Sox11* levels. We propose that lowering *Sox11* levels in mouse mammary tumour cells, particularly tumour cells that are in a hybrid epithelial/mesenchymal state, leads to a release from a non-proliferative state with an increased capacity to expand and undergo invasive growth. Indeed, our results showed that the sg-Sox11-1 cohort displays enhanced proliferation with loss of both epithelial and mesenchymal features, are less differentiated and exhibit an increased metastatic capacity.

High levels of *SOX11* are associated with poor overall survival and increased metastasis in breast cancer patients (Zvelebil et al., 2013). Sox11 regulates a number of similar cellular processes during both normal embryonic development and tumorigenesis, and Sox11-mediated reactivation of embryonic mammary developmental programmes in postnatal breast epithelial cells could lead to the acquisition of features associated with poor patient outcomes. Further investigations into the expression level of SOX11, as well as the expression of its effectors in relation to the regulation of mesenchymal state, multipotency and stemness may lead to ways of stratifying and eventually targeting distinct breast cancer subtypes as SOX11 is associated with an elevated risk of developing metastases and may require more aggressive therapies.

## MATERIALS AND METHODS

### Cell culture

*Brca1*f/f/*p53*±/BLG-Cre (*Brca1^−/−^*) cell lines were a gift from Professor Matthew Smalley (University of Cardiff, UK). eMPC and *Brca1^−/−^* cell lines were cultured using the MesenCult Expansion Kit (mouse) medium (Stem Cell Technologies, 05513) and Dulbecco's modified Eagle medium (DMEM)/F12, respectively, as described previously (Kogata et al., 2018). C2C12, a myoblast cell line, was obtained from the American Type Culture Collection and cultured in DMEM with 10% fetal bovine serum (FBS). The cell lines were screened by Charles River and tested negative for infectious diseases using the mouse essential panel before use in xenograft studies. Karyotyping of eMPC cells was carried out by Cell Guidance Systems. *Brca1*^−/−^ cell lines were authenticated using mouse 384 SNP panel by Charles River.

### Design and construction of CRISPR/Cas9-EGFP plasmids

sgRNAs were designed using the CRISPOR algorithm (www.crispor.tefor.net). sgRNA sequences targeting Sox11 (Gene ID, 20666) were cloned into the Lenti-CRISPR–EGFP plasmid (Addgene, 75159) using a BsmBI enzyme site (sgRNA-1, 5′-TGGACGAGAGCGACCCGGAC-3′; sgRNA-2, 5′-GAGCCCCGACAAGAGCGCGG-3′).

### Transient Neon electroporation

A quantity of 5.0×10^5^ cells was centrifuged at 300 ***g*** for 5 min and resuspended in 100 μl of R1 buffer (Invitrogen). CRISPR/Cas9-EGFP plasmid DNA (10 μg) was added into the cells and loaded into a 100 μl Neon electroporation tip (Invitrogen). Electroporation transfections were performed using 1300 mV for 20 ms with the 2-pulse program on the Neon Electroporator (Invitrogen). After electroporation, cells were cultured in pre-warmed supplemented medium in the plates for 2 days.

### Enrichment of CRISPR/Cas9-targeted cells by flow cytometry

Cells were washed with PBS and harvested with 200 μl fluorescence-activated cell sorting (FACS) buffer [1% bovine serum albumin (BSA) and 0.5 mM EDTA in PBS] 48 h after transfection. A 488-nm diode laser was used for the detection of EGFP. In each sample, viable singlet cells were gated via forward-scatter laser and side-scatter, and EGFP^+^ cells, regardless of expression levels, were sorted using a FACS Aria III flow cytometer (BD Biosciences).

### cDNA synthesis and qRT-PCR

An aliquot of 1 μg of each RNA sample was reverse transcribed using a QuantiTect Reverse Transcription kit (Qiagen) in a final volume of 20 μl. cDNA was diluted tenfold for subsequent qPCR analysis. Comparative *C*_T_ (ΔΔ*C*_T_) qRT-PCR was performed using Power SYBR Green master mix (Applied Biosystems) and a QuantStudio 6 Flex Real-Time PCR System (Applied Biosystems). *Gapdh* was used as an endogenous control and fold-change normalised to a comparator sample was calculated. Primer (Invitrogen) sequences are listed in Table S1.

### Western blot

For Sox11, cells were fractionated into nuclear and cytoplasmic pools using nuclear fractionation protocol from Abcam. Buffer A [10 mM HEPES, 1.5 mM MgCl2, 10 mM KCl, 0.5 mM DTT and 0.05% NP40 (pH 7.9)] was used for the cytoplasmic fraction, whereas Buffer B [5 mM HEPES, 1.5 mM MgCl2, 0.2 mM EDTA, 0.5 mM DTT, 26% glycerol (v/v) (pH 7.9) and 4.6 M NaCl] was used for the nuclear fraction. For other downstream targets, total lysate was collected using RIPA buffer (Upstate) containing 1% NP-40, protease-inhibitor cocktail (Calbiochem) and PhosSTOP (Sigma-Aldrich). The following dilutions of primary antibodies were used for the western blot analysis: 1:500 rabbit anti-Sox11 (Abcam, Ab134107); 1:500 Rab anti-fibronectin (Abcam, Ab2413); 1:500 Rab anti-N-cadherin (Cell Signaling Tachnology, D4R1H); 1:500 rat anti-E-cadherin (Abcam, Ab11512); 1:500 Rab-anti-Mex3A (Abcam, Ab79046); 1:500 Rab anti-Rcor2 (Abcam, Ab37113); 1:1000 Rab anti-GAPDH (Cell Signaling Technology, D16H11); and 1:1000 Rab anti-lamin B1 (Abcam, Ab16048). For detection, an enhanced chemiluminescence detection kit (Bio-Rad) was used.

### Immunofluorescence

Cells were grown as spheres in 96-well low attachment plates for 5 to 7 days before fixation with 4% paraformaldehyde for 20 min at room temperature. Spheres were permeabilised with 0.5% Triton X-100, blocked for 30 min with 0.5% BSA and 0.1% FBS in PBS with 0.1% Triton X-100, and stained with the following primary antibodies: rabbit anti-K14 (Poly19053); rat anti-K8 (TROMA-I, DSHB); and rabbit anti-SMA-Alexa-555 (Ab202509); followed by anti-rabbit IgG Alexa Fluor 488 (Thermo Fisher Scientific, A21206) and anti-mouse IgG Alexa Fluor 555 (Thermo Fisher Scientific, A21203) secondary antibodies and DAPI. All primary and secondary antibodies were used at a 1:500 dilution. DAPI was used at a 1:10,000 dilution. Spheres were mounted in Vectashield (Vector Laboratories) and visualised using 20× magnification on a Leica confocal SP8 microscope. *Z* projection with maximum intensity was used and quantification was performed using the integrated pixel intensity function in ImageJ.

### Fluorescence-activated cell sorting

*Brca1^−/−^*#3 sg-control and sg-Sox11-1 cells were tagged with mCherry/Luc2 and FACS for mCherry and DAPI as described previously (Kogata et al., 2018).

### Spheroid formation assays

Five- to ten-thousand cells were plated in 96-well low attachment plates (Corning 7007, Corning, NY, USA). Medium was replenished every 3-4 days. Images were captured either using an Evos fluorescence microscope or Incucyte. Spheroid morphology was quantified using ImageJ. Relative sphere area was calculated by measuring the sphere area on day 14. For morphogenetic growth (eMPC cells) or invasion assay (*Brca1^−/−^* cells), Matrigel Growth Factor Reduced (BD Biosciences) was added 7 days after plating to a final concentration of 4% and cultured for another 7 days. Relative invasive area was calculated by subtracting the sphere area of day 7 from day 14. For sphere-forming capacity, single cells were grown in the presence of 4% Matrigel in eight-well chambers for 10 days before image analysis. Sphere-forming efficiency was calculated by dividing the number of spheres formed by the number of seeded cells.

### Viability assays

Five-thousand cells were plated in 96-well low attachment plates (7007, Corning, NY, USA) for 5 days. CellTiter–Glo (Promega, Southampton, UK) was used according to the manufacturer's protocol. Luminescence was measured using a Victor X5 58 plate reader (Perkin–Elmer, Seer Green, UK).

### Mammary xenograft studies

Female NSG mice (9-12 weeks) from a colony at the Institute of Cancer Research were housed in ventilated cages on a 12-h light/dark cycle, and received food and water *ad libitum*. All work was carried out under UK Home Office projects and personal licences following receipt of local ethical approval from the Institute of Cancer Research Ethics Committee and in accordance with local and national guidelines.

All mice were randomly selected and 15 mice were allocated to each experimental cohort. *Brca1*^−/−^#3 sg-control and sg-Sox11-1 cells tagged with mCherry/Luc2 were resuspended in equal volume of Matrigel (BD Biosciences, San Diego, CA) before orthotopic injection into the left and/or right inguinal mammary fat pad four of female NSG mice. For metastasis studies, 5×10^5^ mCherry/Luc2-tagged *Brca1*^−/−^#3 sg-control or sg-Sox11-1 cells were implanted orthotopically and primary tumour resection was carried out once the primary tumours reached 8-10 mm in diameter. One sg-control and two sg-Sox11-1 mice were excluded from the study due to unsuccessful removal of primary tumours. For limiting dilution assays, a serial tenfold dilution was used. Animals were weighed and tumours were monitored by caliper measurements and IVIS imaging once a week. Tumour volumes were calculated with the formula: (X.X.Y)/2, where X and Y are the height and width of tumour, respectively. Mice xenografted orthotopically with 1000 cells have a longer tumour latency and were grown to a larger tumour volume compared to the mice injected with 10,000 cells. The tumour-initiating frequency was used for the calculation of the frequency of cancer stem cells using the extreme limiting dilution analysis (ELDA) software web interface (http://bioinf.wehi.edu.au/software/elda). Limiting dilution data were analysed as described previously (Hu and Smyth, 2009).

### Bioluminescence imaging

Secondary tumour regrowth after resection and metastatic tumour development were monitored longitudinally by *in vivo* bioluminescence imaging (BLI) using an IVIS Spectrum Imaging System (PerkinElmer, Waltham, MA, USA) and Living image 4.3.1 software. Mice were injected intraperitoneally with 150 mg/kg D-luciferin (Caliper Life Sciences, Hopkinton, MA, USA) for 5 min before acquisition (exposure time, 5-300 s). BLI signal was quantified as total photon flux (photons/second).

### Immunohistochemistry

Immunohistochemistry was performed on formalin-fixed paraffin-embedded samples. Samples were stained with anti-SOX11 (MRQ-58), K14 and SMA.

### Statistical analysis

Experiments were analysed using a two-tailed Student's *t*-test with a confidence interval of 95% when the number of groups equalled two, unless otherwise specified. Data are mean±s.d.

## Supplementary Material

Supplementary information
