# Supplementary material for: Sox11 regulates mammary tumour-initiating and metastatic capacity in Brca1-deficient mouse mammary tumour cells
Source: Dis Model Mech. 2021 May 10;14(5):dmm046037. doi: 10.1242/dmm.046037 (PMC8188883; doi:10.1242/dmm.046037)
Supplement: Supplementary information [file dmm-14-046037-s1.pdf]

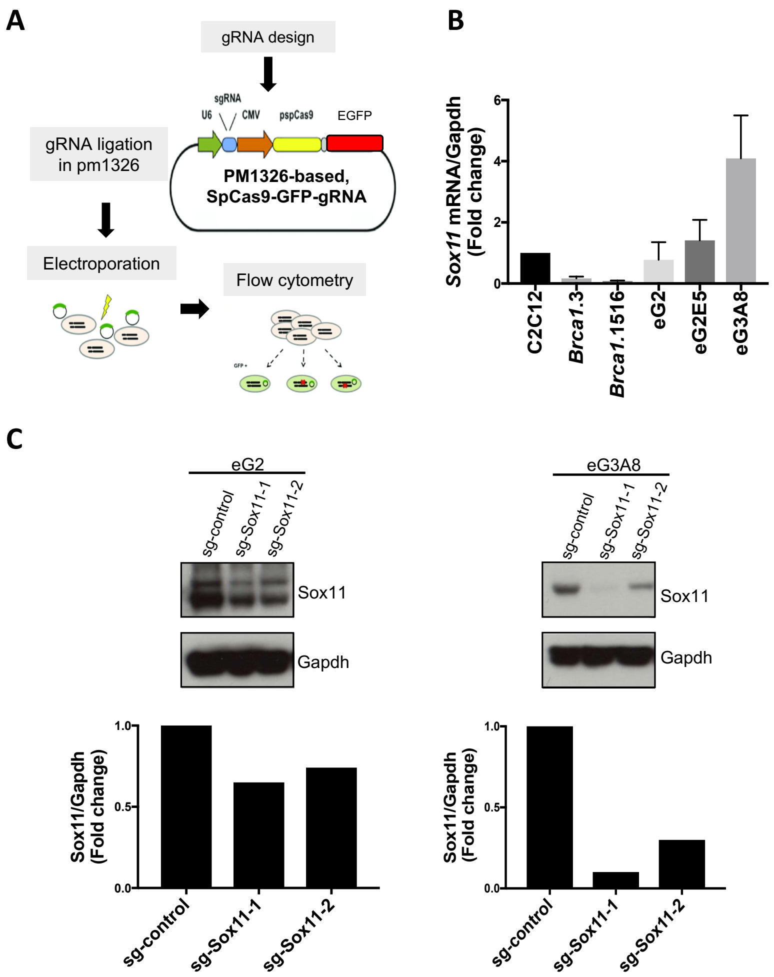

Figure S1.

A. Sox11 CRISPR-CAS9 targeting strategy.

B. qPCR of *Sox11* in C2C12, *Brca1*-/- and eMPC cell lines. N=2 independent experiments. 3 replicates per group per experiment.

C. Western blots and quantification of Sox11 knockdown in two eMPC lines; eG2 (intermediate cluster) and eG3A8 (stem cell-like cluster). eMPC, embryonic mammary progenitor cells.

Disease Models & Mechanisms • Supplementary information

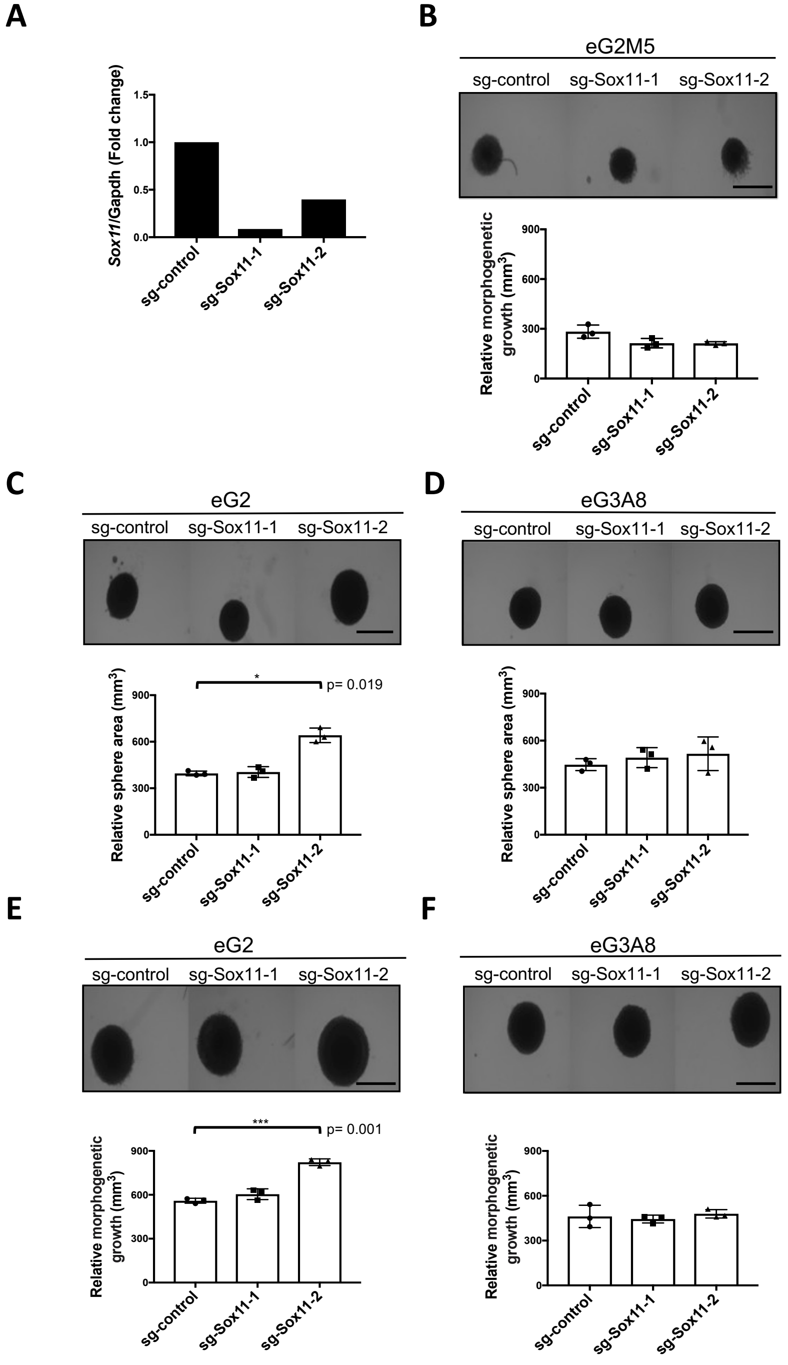

Figure S2.

A. qPCR of *Sox11* in eG2E5 cells after CRISPR targeting of *Sox11*.

B. Morphogenetic growth assay of eG2M5 cells after knockdown of Sox11. eG2E5 cells were grown in low attachment 96-well plates for 7 days before the addition of Matrigel for a further 7 days before image analysis. Relative invasive area was calculated by

Disease Models & Mechanisms • Supplementary information

subtracting the sphere area of day7 from day14. N=3. 3 replicates per group per experiment. Scale bar=1000µm.

C. Sphere formation assay after knockdown of Sox11. eG2 cells were grown in low attachment 96-well plates for 10 days prior to image analysis of relative sphere area. N=3. 2 replicates per group per experiment. Scale bar=1000um.

D. Sphere formation assay of eG3A8 after knockdown of Sox11. N=3. 2 replicates per group per experiment. Scale bar=1000µm.

E. Morphogenetic growth assay of eG2 cells after knockdown of Sox11. N=3. 3 replicates per group per experiment. Scale bar=1000µm.

F. Morphogenetic growth assay of eG3A8 cells after knockdown of Sox11. N=3. 3 replicates per group per experiment. Scale bar=1000µm.

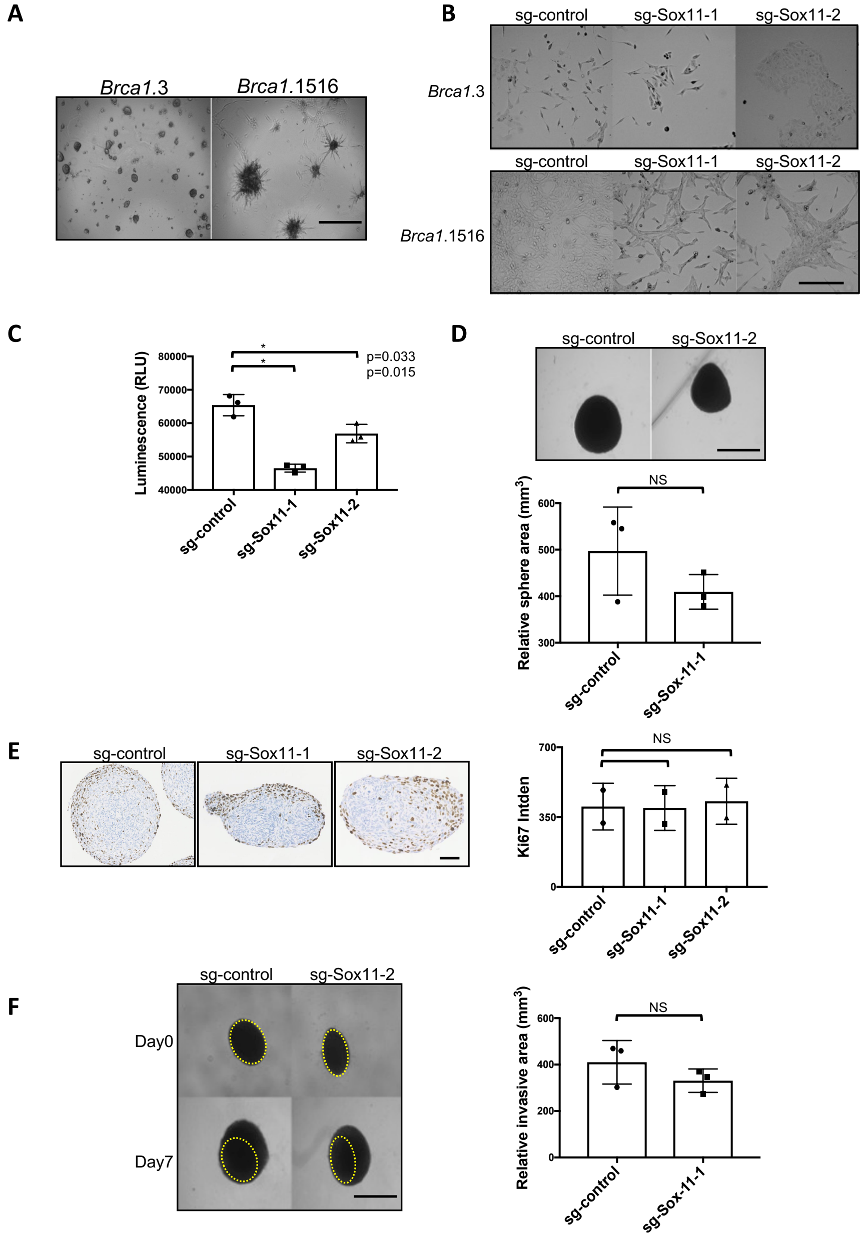

Figure S3.

A. *Brca1*.3 and *Brca1*.1516 spheroid morphology when cultured as single cells in 4% Matrigel. N=3 independent experiments. 2 replicates per group per experiment. Scale= 1000µm.

B. Phenotypes of *Brca1*.3 and *Brca1*.1516 cells culture in 2D with Sox11 knockdown. Scale bar=400µm.

Disease Models & Mechanisms • Supplementary information

C. Cell viability after knockdown of Sox11 in *Brca1.*1516 cells as determined by CellTiter-Glo assay. N=3 independent experiments. 7 replicates per group per experiment.

D. Sphere formation assay after knockdown of Sox11. *Brca1.*1516 cells were grown in low attachment 96-well plates for 10 days prior to image analysis of relative sphere area. N=3 independent experiments. 4 replicates per group per experiment. Scale bar=1000μm.

E. Quantification of Ki67 expressing-cells as compared to control. N=3 independent experiment. Minimal 2 replicates per group per experiment. Scale bar=200μm.

F. 3D invasion assay of *Brca1.*1516 cells after knockdown of Sox11. Relative invasive area was calculated by subtracting the sphere area of day7 from day14. N=3 independent experiments. 4 replicates per group per experiment. Scale bar=1000μm.

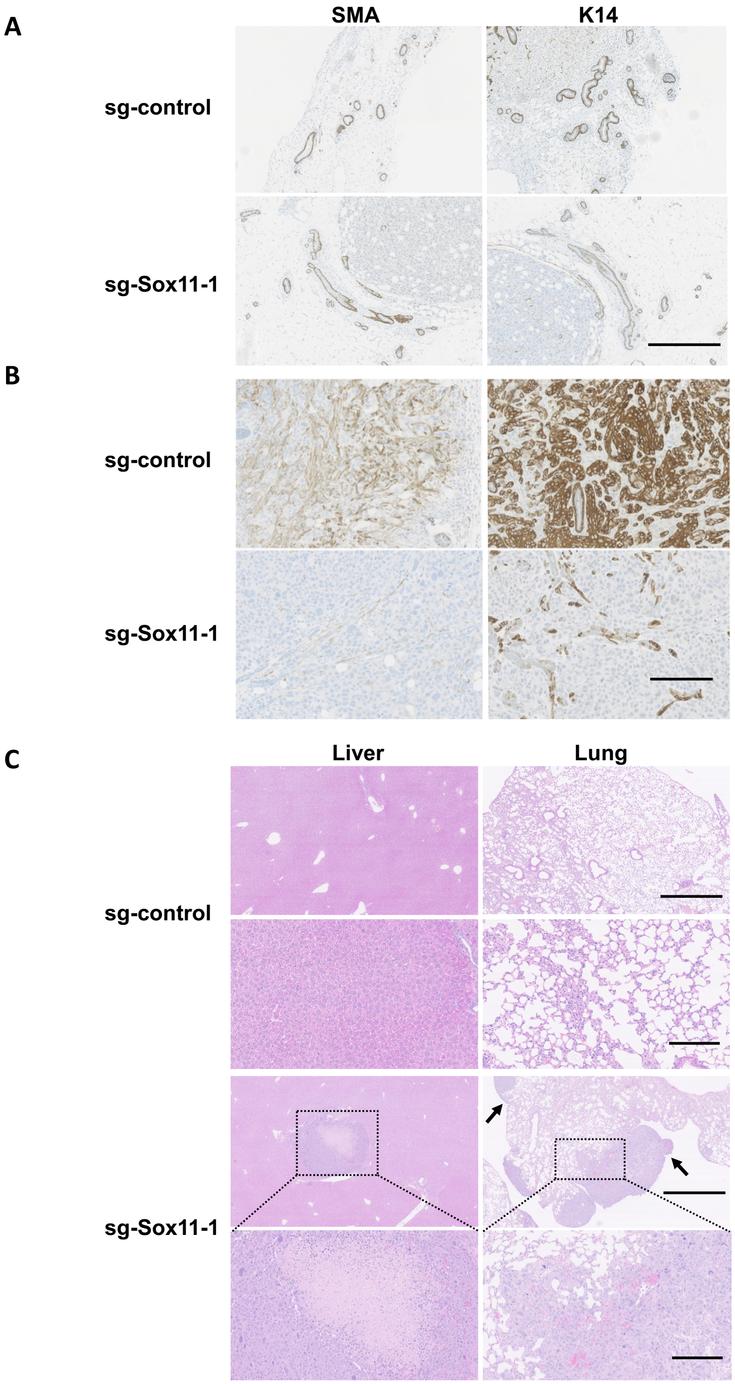

Figure S4.

A. Results from immunohistochemistry of primary tumours stained with markers SMA and K14. Internal positive controls demonstrating both sg-control and sg-Sox11-1 tumours were positive for both K14 and SMA in the normal mammary tissue adjacent to the tumour. Scale bar=500μm.

Disease Models & Mechanisms • Supplementary information

B. Higher magnification of sg-control and sg-Sox11-1 tumours stained with K14 and SMA by IHC. Scale bar=200µm.

C. H&E of *Brca1*.3 liver and lung metastasis. Black arrows point to the lung nodules. Scale bar= 200µm and 500um.

Table S1. Primer sequences for qRT-PCR

	Primer sequences
Sox11 (mouse)	5′-AAGAACATCACCAAGCAGCA-3′
	5′-TCCAGGTCCTTATCCCACCAG-3′
Gapdh (mouse)	5′-CGACTTCAACAGCAACTCCCACTCTTCC-3′
	5′-TGGGTGGTCCAGGGTTTCTTACTCCTT-3′

Disease Models & Mechanisms • Supplementary information
